# Predicting Fibrosis Stage in MASH: The Role of Total Metabolic Syndrome Score and MMP-1

**DOI:** 10.3390/medicina61061102

**Published:** 2025-06-17

**Authors:** Bahadır Köylü, Cenk Sökmensüer, Muşturay Karçaaltıncaba, Onur Keskin

**Affiliations:** 1 Department of Internal Medicine, Faculty of Medicine, Hacettepe University, 06230 Ankara, Turkey; 2 Department of Pathology, Faculty of Medicine, Hacettepe University, 06230 Ankara, Turkey; cenksokmensuer@hacettepe.edu.tr; 3 Department of Radiology, Faculty of Medicine, Hacettepe University, 06230 Ankara, Turkey; mkarcaal@hacettepe.edu.tr; 4 Division of Gastroenterology, Department of Internal Medicine, Faculty of Medicine, Hacettepe University, 06230 Ankara, Turkey; onurkeskin@hacettepe.edu.tr

**Keywords:** fatty liver, biomarker, magnetic resonance elastography, liver fibrosis

## Abstract

*Background and Objectives*: Fibrosis stage is the key histopathological determinant of liver-related outcomes in metabolic dysfunction-associated steatohepatitis (MASH); however, a reliable noninvasive method for predicting fibrosis stage remains an unmet need. This study aimed to develop an accurate, practical, and noninvasive tool for identifying “at-risk MASH patients”. *Materials and Methods*: Fifty-six patients with biopsy-confirmed MASH were prospectively enrolled and categorized into fibrosis stages using the NASH-CRN system. In addition to anthropometric and biochemical parameters, seven serum fibrosis biomarkers were evaluated across fibrosis stages. Binary logistic regression analysis was used to construct a scoring model for predicting ≥F2 fibrosis. The diagnostic performance of the proposed model was compared with established noninvasive tests (NITs) and magnetic resonance elastography (MRE) for detecting both ≥F2 and ≥F3 fibrosis. *Results*: The total metabolic syndrome score was the only variable that significantly distinguished between F1 and F2 stages (*p* = 0.039). Among the biomarkers, matrix metalloproteinase-1 (MMP-1) showed a significant difference across fibrosis groups (*p* = 0.009). The AST/ALT ratio was the most robust predictor for differentiating ≥F3 (*p* < 0.001). A scoring model integrating the total metabolic syndrome score, MMP-1, and AST/ALT ratio demonstrated superior diagnostic accuracy for identifying ≥F2 (AUROC 0.88, 95% CI 0.79–0.97) compared to other NITs and MRE, and strong performance for detecting ≥F3 (AUROC 0.95, 95% CI 0.90–1.00). *Conclusions*: Total metabolic syndrome score and MMP-1 are promising candidates for future approaches. Combining total metabolic syndrome score, MMP-1, and AST/ALT ratio might detect ≥F2 in MASH with higher diagnostic accuracy than other NITs and MRE.

## 1. Introduction

The current terminology of metabolic dysfunction-associated steatotic liver disease (MASLD) has replaced that of nonalcoholic fatty liver disease (NAFLD), and the use of NAFLD term is now discouraged [1]. Although MASLD and NAFLD involve a change in definition, recent studies have shown that there is a near-complete overlap between MASLD and NAFLD patients in terms of prevalence, clinical profiles, outcomes, and efficacy of diagnostic biomarkers [2,3]. These findings provide evidence that NAFLD data gathered to date remain valid for future MASLD studies. Recently, MASLD has become the leading cause of chronic liver disease worldwide, with an estimated prevalence of 30% [4]. Within this spectrum, metabolic dysfunction-associated steatohepatitis (MASH) represents the more aggressive phenotype [5]. Recent studies suggest that MASH patients with fibrosis stage ≥F2 constitute the main risk group for liver-related outcomes [6,7,8]. Resmetirom has been recently approved by the U.S. Food and Drug Administration (FDA) as the first therapeutic agent targeting this high-risk subgroup [9,10]. As new agents emerge, accurate staging of fibrosis will remain essential to guide treatment decisions [11].

Despite advances, a reliable and broadly accessible noninvasive method for fibrosis staging in MASH remains an unmet need. Liver biopsy, the current gold standard for diagnosing MASH and staging fibrosis, has several key limitations, including high sampling error rate, intra and interobserver variability in pathological assessment, cost, and potential complications [12]. High-quality imaging methods are not always accessible or affordable, especially in low-resource regions, whereas suboptimal imaging methods might not yield reliable results for clinical decisions [13]. The primary aim of this study is to identify significant clinical predictors of fibrosis stage in a well-defined MASH cohort. The cohort is validated using liver biopsy, magnetic resonance elastography (MRE), and magnetic resonance imaging-proton density fat fraction (MRI-PDFF). Furthermore, we aim to develop an accurate, widely implementable, noninvasive predictive model by integrating clinical variables and serum fibrosis biomarkers, with a specific focus on identifying patients with fibrosis stage ≥F2, regarded as the high-risk group.

## 2. Materials and Methods

### 2.1. Study Design

This study prospectively enrolled 56 biopsy-confirmed MASH patients from January 2020 to January 2021, before the terminology was updated. A review of their clinical characteristics through the database showed that all patients had at least one cardiometabolic risk factor, consistent with the current MASH definition, with 10 patients having two and 41 patients having at least three cardiometabolic risk factors. During the enrolment period, only patients with a biopsy-confirmed diagnosis of MASH within one year were enrolled and deemed eligible for subsequent evaluations, including anthropometric assessments, laboratory testing, and imaging procedures. Patients who applied to Hacettepe University Department of Gastroenterology with elevated liver enzymes and diagnosed with MASH by liver biopsy were carefully evaluated with history, physical examination, anthropometric measurements, biochemical tests, and imaging MRE and MRI-PDFF. Other causes of elevated liver enzymes were excluded carefully (see inclusion and exclusion criteria section). The patients enrolled in the study were divided into groups based on the pathological fibrosis stages. Since no patient had stage 0 fibrosis (F0), patients were classified into four groups as stage 1–4 fibrosis (F1–4). Demographic data, anthropometric measurements, biochemical data, serum fibrosis biomarkers, pre-biopsy scores of noninvasive tests (Fibrosis-4 [FIB4] [14], NAFLD fibrosis score [NFS] [15], BARD score [16], and AST to platelet ratio index [APRI] [17]), and MRE kilopascal (kPa) measurements were compared according to this classification. Patients with ≥F2 were classified as “at-risk MASH patients”. Binary logistic regression analysis was performed based on this grouping for the development of the new scoring model. Additionally, patients in F3 and F4 groups were classified as “advanced fibrosis”. The diagnostic accuracies of the new scoring model, FIB4, NFS, BARD score, APRI score, and MRE kPa measurements for the detection of both “at-risk MASH patients”(≥F2) and “advanced fibrosis”(≥F3) were compared with the ROC analyses.

This study was approved by Hacettepe University Ethics Committee (Approval ID: GO 19/1148, 7 January 2020). All patients provided written informed consent prior to enrolment.

### 2.2. Inclusion and Exclusion Criteria

Only patients aged 18–75 years and diagnosed by percutaneous liver biopsy within one year of the study enrolment were included. Patients were excluded in the presence of other causes of liver disease (hepatitis B and C, autoimmune hepatitis, drug-induced liver injury, Wilson’s disease, hemochromatosis), in the presence of secondary causes of hepatic steatosis, including alcohol ingestion (>30 g per day for men, >20 g per day for women) and long-term use of steatogenic drugs (e.g., corticosteroids, tamoxifen, methotrexate), in the presence of severe comorbidities (e.g., heart failure, renal failure, malignancies), and pregnancy.

### 2.3. Anthropometric Measurements

Body height, waist circumference, and hip circumference of each patient were measured. The waist circumference was measured parallel to the ground at the level of upper boundary of the crista iliaca. Hip circumference was measured at the widest point of the hips, parallel to the ground. To ensure standardization, all measurements were taken using the same measuring instrument and by the same person. Weight measurements and segmented body analysis were conducted using the TANITA BC 418 MA device (Tanita Corporation, Tokyo, Japan), following a minimum of 8 h of fasting. All anthropometric measurements were taken for each patient on the same day that serum samples were collected from that patient.

### 2.4. Biochemical Tests for Serum Fibrosis Biomarkers

For the analyses of serum fibrosis biomarkers (alpha-2 macroglobulin [A2M], apolipoprotein A1 [ApoA1], hyaluronic acid, tissue inhibitor of matrix metalloproteinase [TIMP-1], procollagen III N-terminal propeptide [PIIINP], matrix metalloproteinase-1 [MMP-1], and MMP-3), blood samples of participants were collected in the morning, following a minimum of 8 h of fasting. The samples were stored in −80 °C freezer. All samples were analyzed using sandwich ELISA with commercially available kits (Bioassay Technology Laboratory, Shanghai, China: E1097Hu, E1535Hu, E1374Hu, E6276Hu, E1353Hu, E0916Hu, E0907Hu). The following laboratory tests were also conducted and included in the study: alanine aminotransferase (ALT), aspartate aminotransferase (AST), alkaline phosphatase (ALP), gamma-glutamyl transferase (GGT), total bilirubin, direct bilirubin, indirect bilirubin, platelet count, international normalized ratio (INR), activated partial thromboplastin time (aPTT), albumin, blood urea nitrogen (BUN), glycated hemoglobin (HbA1c), fasting plasma glucose level, homeostatic model assessment for insulin resistance (HOMA-IR), total cholesterol, low-density lipoprotein (LDL), high-density lipoprotein (HDL), non-HDL, triglyceride, haptoglobin, ammonia (NH3), α-fetoprotein, and 25-hydroxyvitamin D_3_ (25[OH]D_3_).

We also introduced a novel variable, the “Total Metabolic Syndrome Score”, which combines anthropometric measurements and biochemical tests. It is calculated by assigning one point for each of the five criteria used in the diagnosis of metabolic syndrome, yielding a total score ranging from 0 to 5, based on the NCEP ATP III (National Cholesterol Education Program Adult Treatment Panel III) guidelines [18] (Supplementary Table S1).

### 2.5. Histological Evaluation

All liver biopsy specimens were systematically reanalyzed by an expert liver pathologist (C.S.) who was unaware of clinical and imaging data. The NASH-CRN Histologic Scoring System was used during the histological assessment [19]. The NAFLD Activity Score (NAS), ranging from 0 to 8, was obtained by summing the steatosis (0 to 2), lobular inflammation (0 to 3), and hepatocellular ballooning (0 to 3) scores. MASH was defined by the presence of hepatic steatosis with lobular inflammation and definite hepatocellular ballooning with or without fibrosis. Fibrosis was staged from 0 to 4 (F0: No fibrosis; F1: Either mild-moderate peri-sinusoidal or peri-portal fibrosis; F2: Both peri-sinusoidal and portal/peri-portal fibrosis; F3: Bridging fibrosis; F4: Cirrhosis) [19].

### 2.6. Magnetic Resonance Elastography and Proton Density Fat Fraction

The radiologist (M.K.) who performed the MRE kPa and MRI-PDFF measurements was blinded to clinical and histopathological data. MRI was performed with a 1.5-T MR system (Magnetom Aera, Siemens Healthcare, Erlangen, Germany). A 30-channel phased-array body coil was used for this acquisition. A three-plane localization imaging gradient echo sequence was performed at the beginning of the examination. The MRE parameters were as follows: TR/TE 50 ms/21.41 ms, flip angle 25°, section thickness 50 mm, and field of view (FOV) 350 × 350 mm^2^ with a passive driver frequency of 60 Hz. Using a workstation (Syngo.via VB10; Siemens Medical Solutions, Erlangen, Germany), regions of interest (ROI) were drawn as geographic areas guided by the magnitude image to include liver parenchyma by excluding major vessels. The multi-echo Dixon VIBE sequence (Siemens Healthcare, Erlangen, Germany) was used with the following parameters: repetition time 15.6 ms, 6 echo times (1.23, 2.48, 3.73, 4.98, 6.23, and 7.48 ms), flip angle 4°, readout echo bandwidth 1080 Hz/pixel, FOV 450 mm, and slice thickness 3.5 mm. Using a Workstation (Syngo.via VB10; Siemens Medical Solutions, Erlangen, Germany), PDFF values were calculated.

### 2.7. Statistical Analysis

IBM^®^ SPSS^®^ Statistics 24.0 (IBM Corporation, Armonk, NY, USA) and EasyROC version 1.3.1 (BioSoft, Erciyes University, Kayseri, Turkey) were used to conduct the analysis. The normality of the data was evaluated through visual methods (histograms and probability graphs) and the Shapiro–Wilk test. Descriptive statistics for continuous variables were presented as median and interquartile range (IQR) since they were not normally distributed. Descriptive statistics for dichotomous variables were presented as numbers and percentages. The comparisons between the four independent groups for dichotomous variables were assessed by Pearson’s chi-squared test, and Bonferroni correction was applied in post hoc analyses for variables that reached statistical significance. For continuous variables, the Kruskal–Wallis test was used to compare the four independent groups, and the Dunn–Bonferroni method was used for post hoc analyses. Continuous and dichotomous variables that exhibited statistically significant differences in comparisons among the four independent fibrosis groups were identified as “candidate variables” for the intended new scoring model. Binary logistic regression analysis was performed on these candidate variables to create a new scoring model. The diagnostic accuracies of the proposed scoring model, noninvasive tests (NITs), and MRE kPa for stage ≥2 fibrosis (≥F2) and stage ≥3 fibrosis (≥F3) were evaluated by ROC analyses, calculating the area under the ROC curve (AUROC), the optimal thresholds, positive predictive value (PPV), and negative predictive value (NPV). Youden’s index was used to determine the optimal thresholds. The DeLong test was used to compare the AUROCs. A two-tailed *p* value <0.05 was considered statistically significant.

## 3. Results

A total of 56 patients were included, of whom 48.2% were female. Imaging data from MRE and MRI-PDFF were available for 49 patients (87.5%). The median (IQR) time intervals between liver biopsy and serum sample collection, liver biopsy and MRI, and MRI and serum collection were 25.5 (13–101), 18 (7.5–45.5), and 7 (2–43) days, respectively. Histopathological evaluation revealed no patients with stage 0 fibrosis (F0). The distribution of fibrosis stages was as follows: F1 in 23 patients (41.1%), F2 in 19 (33.9%), F3 in 6 (10.7%), and F4 in 8 (14.3%).

**Figure 1:**
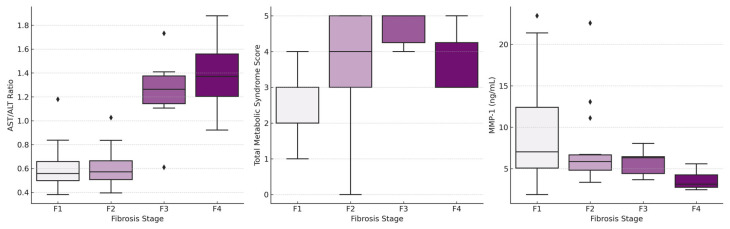
Bar graphs of the variables included in the proposed scoring model according to liver fibrosis stages.

In the comparison between fibrosis groups for demographic variables, statistically significant differences were found for age, gender, presence of metabolic syndrome, and total metabolic syndrome score (Table 1). The median age increased gradually as the fibrosis stage progressed (*p* = 0.001). A statistically significant difference in the gender was observed among the groups (*p* = 0.042). Post hoc analyses revealed that this difference was due to all patients in the F3 group being female. The difference among the groups in terms of the presence of metabolic syndrome was attributed to a markedly lower rate of metabolic syndrome in F1 group compared to other groups, and the presence of metabolic syndrome in all patients of F4 group (*p* = 0.011). Total metabolic syndrome score was also lower in F1 group compared to F2 and F3 groups (*p* = 0.039 for F1–F2, *p* = 0.001 for F1–F3) (Figure 1). No statistically significant difference was detected between the groups in the analyses of blood pressure measurements and anthropometric data (Supplementary Table S2).

The biochemical variables that reached statistical significance were ALT, AST/ALT ratio, platelet count, INR, albumin, BUN, fasting plasma glucose, and LDL (Table 1). The AST/ALT ratio was the most discriminative variable for differentiating fibrosis stage ≥F3 (*p* < 0.001). As the fibrosis stage progressed, the AST/ALT ratio was substantially higher in F3 and F4 groups compared to F1 and F2 groups (Figure 1). There was no difference between F1–F2 and F3–F4 groups, but statistically significant differences were found in all other pairwise comparisons (*p* = 0.005 for F1–F3, *p* < 0.001 for F1–F4, *p* = 0.011 for F2–F3, *p* < 0.001 for F2–F4). Among the serum fibrosis biomarkers, the only molecule that reached statistical significance was MMP-1 (*p* = 0.009) (Table 2). The median (IQR) value for MMP-1 in F1 group was 7.03 ng/mL (5.05–12.90), while the median (IQR) values were 5.85 ng/mL (4.68–6.71), 6.31 ng/mL (3.82–6.44), and 3.13 ng/mL (2.72–4.29) in F2, F3, and F4 group, respectively. The progression of the fibrosis stage was associated with a trend of decreasing serum MMP-1 levels (Figure 1). Post hoc analyses unveiled that this difference for MMP-1 stemmed from the notable differences between F1–F4 and F2–F4 groups (*p* = 0.004 for F1–F4, *p* = 0.048 for F2–F4).

All NITs showed statistically significant differences across fibrosis stages (*p* < 0.001 for FIB4; *p* < 0.001 for NFS; *p* < 0.001 for BARD score; *p* = 0.005 for APRI score) (Table 2). MRE kPA measurements increased with advancing fibrosis stage. The median (IQR) values for MRE kPA were 2.50 kPa (2.40–2.90), 2.70 kPa (2.20–3.50), 4.75 kPa (2.95–6.50), and 6.00 kPa (4.00–7.00) in F1, F2, F3, and F4 groups, respectively (*p* < 0.001). The correlation between fibrosis stage and MRE kPa measurement was moderate (*r* = 0.55, *p* < 0.001). A strong correlation was observed between the percentage of histological steatosis and MRI-PDFF measurement (*r* = 0.79, *p* < 0.001).

By using candidate variables, binary logistic regression analysis was performed to develop a scoring model that could identify MASH patients with ≥F2. The final equation consisting of three parameters (AST/ALT ratio, MMP-1 level, and total metabolic syndrome score) is as follows:

Proposed Scoring Model = −4.414 + (2.830 × AST/ALT ratio) − (0.049 × MMP-1[ng/mL]) + (0.959 × Total Metabolic Syndrome Score)

**Figure 2:**
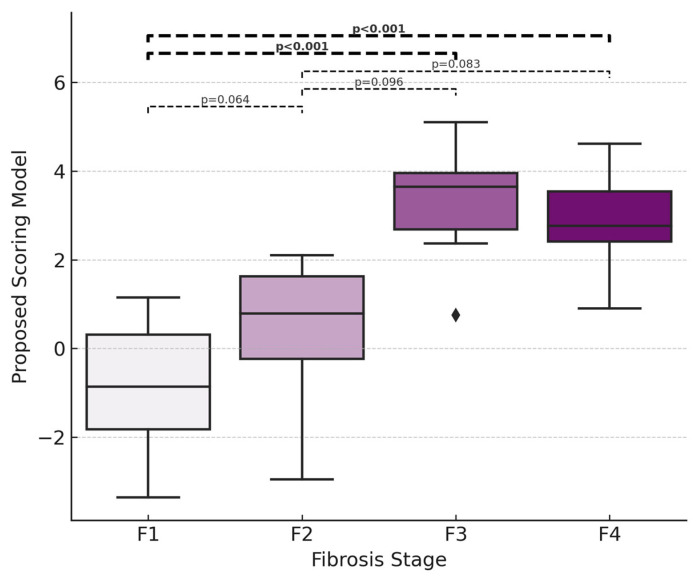
Comparison of the results of the proposed scoring model according to liver fibrosis stages.

The median (IQR) values for the proposed scoring model were −0.86 (−1.83–0.38), 0.78 (−0.24–1.62), 3.65 (2.37–4.05), and 2.76 (2.17–3.57) in F1, F2, F3, and F4 groups, respectively *(p* < 0.001) (Figure 2). Post hoc analyses established that this difference stemmed from the marked differences between F1–F3 and F1–F4 groups (*p* < 0.001 for F1–F3, *p* < 0.001 for F1–F4). Although there was a progressive increase in the proposed scoring model as the fibrosis stage increased from F1 to F3, no statistically significant difference was found between F1–F2 and F2–F3 groups (*p* = 0.064 for F1–F2, *p* = 0.096 for F2–F3, *p* = 0.083 for F2–F4).

**Figure 3:**
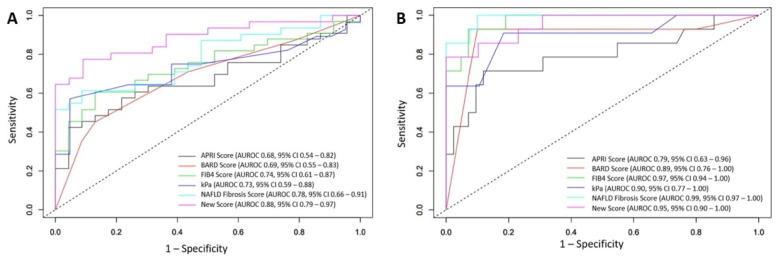
(**A**) Comparison of diagnostic accuracies of the proposed new score, FIB4, NFS, BARD score, APRI score, and MRE kPa measurements with ROC analysis for ≥F2 and (**B**) for ≥F3.

The proposed scoring model had a higher AUROC value (AUROC 0.88, 95% CI 0.79–0.97) compared to other NITs and MRE kPa in predicting patients with ≥F2 (Figure 3A). The AUROC for the proposed scoring model was statistically significant compared to the BARD score (*p* = 0.0024), APRI score (*p* = 0.014), and MRE kPA (*p* = 0.03), but it was not significant compared to FIB4 (*p =* 0.097) and NFS (*p =* 0.089). Using a threshold of 0.75, the proposed scoring model achieved a PPV of 92.3% in diagnosing ≥F2, and also had a higher sensitivity (77.4%) and NPV (74.1%) compared to other NITs and MRE kPa (Table 3). AUROC value of the proposed scoring model for the diagnosis of advanced fibrosis (≥F3) was found to be 0.95 (95% CI 0.90–1.00), which was comparable to the AUROC values of FIB4 (AUROC 0.97, 95% CI 0.94–1.00) and NFS (AUROC 0.99, 95% CI 0.97–1.00) (Figure 3B). The AUROC for the proposed scoring model was statistically significant compared to MRE kPA (*p* = 0.0063). Although the proposed scoring model had a lower sensitivity compared to other NITs and MRE kPa, it yielded the highest specificity (100%) and PPV (100%) in diagnosing advanced fibrosis (≥F3) by using the calculated optimal cut-off value of 2.37. These findings support the potential utility of the proposed scoring model as a practical noninvasive tool for fibrosis staging in MASH.

## 4. Discussion

This study is one of the first to include all patients based on the new terminology, with liver biopsy confirmation for all, the majority undergoing MRE and MRI-PDFF imaging, and a comprehensive analysis of serum fibrosis biomarkers. The proposed scoring model offers higher diagnostic accuracy for identifying “at-risk MASH patients” (≥F2) compared to other NITs and MRE kPa measurements. It also provides strong diagnostic accuracy in detecting patients with advanced fibrosis (≥F3).

Previous studies have highlighted that the key histopathological determinant of liver-related outcomes in MASH is the stage of fibrosis, while the severity of steatosis and inflammation does not have a significant impact on prognosis [8,20]. To assess the severity of fibrosis in MASH patients, NITs are easily accessible, cost-effective, and safe methods. NITs derived from commonly used variables in clinical practice have demonstrated effectiveness, particularly in detecting advanced fibrosis (≥F3) [21,22]. FIB4, NFS, BARD score, and APRI score are widely used NITs in clinical practice to stratify fibrosis risk. Since these scores are based on readily available clinical and laboratory parameters, they are practical tools for routine screening and risk stratification. Among them, FIB4 and NFS have been extensively validated and are recommended by major guidelines as first-line tools for risk stratification [23,24]. However, these NITs fall short in approximately 30% of cases by generating results within an indeterminate gray zone, thereby restricting their effectiveness in guiding clinical decision-making [21,22]. In our study, NFS and FIB4 scores effectively identified patients with advanced fibrosis with high diagnostic accuracy. However, these tests performed better in excluding advanced fibrosis, as all NITs had higher NPVs than PPVs in our study. More importantly, these NITs failed to reliably distinguish patients in F2 group, the largest subgroup of “at-risk MASH patients”. In various clinical trials, patients with ≥F2 fibrosis have been defined as “at-risk MASH patients” [25]. In our study, ROC analyses for patients with ≥F2 showed a significant decline in the diagnostic accuracies of FIB4, NFS, BARD score, and APRI score compared to ≥F3. Furthermore, these NITs were found to have considerably low sensitivities and NPVs, indicating that they are not reliable in detecting patients in the F2 group, which represents over 50% of “at-risk MASH patients” and poses a significant risk for fibrosis progression and liver-related complications [26]. To better differentiate the F2 group from the ≤F1 group and improve the accuracy of identifying “at-risk MASH patients”, our findings support incorporating the total metabolic syndrome score into future approaches, as it was the only variable that showed a statistically significant difference between the F1 and F2 groups.

The link between metabolic syndrome and MASLD is well established [27]. In metabolic syndrome, insulin resistance precipitates hepatic lipotoxicity and oxidative stress, leading to hepatocellular injury that triggers chronic inflammation through Kupffer cell activation and monocyte infiltration [28]. Concomitantly, dysregulated adipokine release from dysfunctional adipose tissue amplifies profibrotic signaling [28]. The interplay of these mechanisms promotes hepatic stellate cell activation and progressive fibrosis through excessive extracellular matrix (ECM) accumulation [28]. In a recent longitudinal study, Wu et al. demonstrated that MASLD onset is significantly correlated with alterations in metabolic syndrome components [29]. Similarly, DeBoer et al., in a retrospective analysis, reported that each standard deviation increase in the metabolic syndrome severity Z-score (MetS-Z) was associated with a greater likelihood of elevated ALT (OR = 1.58, 95% CI 1.44–1.72) and advanced fibrosis (OR = 1.96, 95% CI 1.77–2.18) [30]. Notably, this study relied on elevated ALT and NFS, rather than liver biopsy, to define NAFLD and advanced fibrosis, respectively. In contrast, our study revealed that the total metabolic syndrome score was significantly lower in patients with biopsy-confirmed early-stage fibrosis than in those with ≥F2 fibrosis. As this score can be easily calculated from routine laboratory and physical examination data, it may serve as a valuable indicator of metabolic burden. While equal weighting was used for simplicity and alignment with existing criteria, it is possible that specific components of metabolic syndrome contribute more strongly to fibrosis progression. This warrants further investigation and potential adjustment in future research. Nonetheless, combining the total metabolic syndrome score with the AST/ALT ratio, a simple and reliable indicator of advanced fibrosis [27,31,32], offers significant potential to improve noninvasive assessment of fibrosis severity.

Advanced scoring models incorporating various molecules linked to hepatic fibrogenesis may offer greater accuracy in predicting fibrosis stage than easily calculable models [33]. Our study found that, among the seven serum fibrosis biomarkers analyzed, only MMP-1 exhibited a significant difference between the groups, demonstrating a trend of decreasing levels with fibrosis stage progression. Matrix metalloproteinases (MMPs) exhibit a dynamic role in regulating ECM remodeling during the course of liver fibrosis, with their impact varying according to temporal expression patterns and activity levels [34]. MMP-1 is the major interstitial collagenase in humans [35]. MMP-1 facilitates the proteolytic degradation of ECM components, including collagen, gelatin, and laminin, thereby promoting antifibrotic activity and attenuating the progression of fibrosis [34,35]. In animal studies, transient overexpression of MMP-1, which is capable of degrading collagen types I and III—the primary collagens in irreversible scar tissue—has been shown to effectively reduce hepatic fibrosis and promote hepatocyte proliferation [36]. Several studies have reported a negative correlation between serum MMP-1 levels and the stage of hepatic fibrosis in chronic liver diseases [35,37]. Furthermore, MMP-1 expression was found to be increased in hepatic progenitor cells from the early stages of nonalcoholic steatohepatitis (NASH), likely due to inflammation and steatosis [38]. Notably, patients with early-stage fibrosis (F1) had significantly higher serum MMP-1 levels compared to those with more advanced fibrosis stages and healthy controls [38]. These findings provide a strong rationale to further investigate the potential role of MMP-1 for noninvasive fibrosis staging in MASH. In contrast, another study found TIMP-1 to be the only independent predictor of fibrosis in NAFLD patients, with no significant difference observed for MMP-1 [39]. A study assessing serum fibrosis biomarkers for staging fibrosis in chronic hepatitis C patients found that only MMP-1 and PIIINP were independently associated with fibrosis, leading to the development of the MP3 score based on their combination [40]. Despite its promising ability to distinguish early-stage fibrosis, MMP-1 has not yet been integrated into any validated scoring models for MASH patients.

MRE is an imaging modality that measures liver stiffness and has been shown to have significantly higher diagnostic accuracy for hepatic fibrosis, even in the early stages, compared to serum fibrosis biomarkers [21,41]. It is a promising modality as a sort of “virtual biopsy” for MASLD patients, since it may significantly decrease the need for invasive liver biopsy, as well as its related complications and costs [42]. While the suboptimal reliability of liver biopsy is a major concern due to sampling errors and inter-/intra-observer variability in the pathological assessment, MRE/MRI-PDFF is highly reproducible and MRE may eliminate the risk of understaging fibrosis by liver biopsy in patients with high spatial heterogeneity in liver stiffness [43,44]. Despite these potential benefits, MRE/MRI-PDFF is an expensive and time-consuming method, limiting its extensive use. In our study, the optimal threshold value of MRE kPa calculated for advanced fibrosis, 3.50 kPa, is consistent with previous studies [45,46]. The optimal threshold value for patients with ≥F2 was also calculated as 3.50 kPa, slightly higher than previous studies [46]. The identical cut-off values arise from the substantial overlap in MRE kPa measurements between the F1 and F2 groups, as well as the relatively wide range of MRE kPa measurements in the F2 group, which also overlaps with the F3 group. Although high diagnostic accuracy and high NPV for advanced fibrosis were achieved with MRE kPa measurements, the PPV of 58.8% indicates a significant number of false positive results, arising mostly from overlapping patients in the F2 group. The PPV for ≥F2 was 94.1%, while the NPV and sensitivity decreased to 62.5% and 57.1%, respectively. This decline is due to the fact that a portion of patients in F2 group have MRE kPa measurements >3.50 kPa, while a significant number of patients have measurements <3.50 kPa. Therefore, when MRE is used alone for the detection of patients with ≥F2, there is a considerable risk of misclassifying a huge number of patients in the F2 group as false negatives.

Currently, the investigation of miscellaneous possible diagnostic and/or prognostic noninvasive biomarkers is in progress [47]. However, there are few NITs specifically designed and validated to detect ≥F2 in MASH. Available NITs typically have greater specificity and high NPV for ≥F3, while the PPV is modest as the prevalence of advanced fibrosis is low. They are less successful in predicting ≥F2 [48]. Until today, limited research has been conducted to compare the diagnostic accuracies of NITs and MRE kPa for the noninvasive diagnosis of ≥F2 in MASH patients. A major advantage of our study is that a significant number of patients underwent MRE/MRI-PDFF, enabling comparisons of MRE kPa measurements with the proposed new scoring model and other NITs. Furthermore, various relevant fibrosis biomarkers were comprehensively analyzed and compared across fibrosis groups. The relatively short intervals between liver biopsy, serum sampling, and MRE imaging further enhance the strength and value of this comparative analysis. The total metabolic syndrome score and MMP-1 levels, two variables not currently included in NITs for MASH, have emerged as promising candidates for future approaches, alongside the widely used AST/ALT ratio. With a significantly high PPV, as well as higher sensitivity and NPV compared to other NITs and MRE kPa, the proposed scoring model offers a simple, cost-effective, and broadly applicable tool for both screening and follow-up of ≥F2 fibrosis, potentially reducing the need for liver biopsy and MRE in routine clinical practice. However, our study has some limitations. First, the limited number of patients in each group and single-center design reduce the robustness of the data. Despite enrolling all biopsy-proven MASH patients within a defined period at our clinic, the infrequent use of liver biopsy in routine clinical practice, along with the COVID-19 pandemic during the enrolment period, restricted our ability to recruit more participants. Additionally, in clinical practice, liver biopsy is rarely performed in patients with possible advanced fibrosis due to the increased risk of bleeding associated with thrombocytopenia and coagulopathy. Consequently, the number of biopsy-confirmed patients in the F3 and F4 fibrosis groups is lower compared to earlier stages. However, the primary objective of our study was to reliably and noninvasively differentiate patients with “at-risk MASH,” defined as those with fibrosis stage ≥F2, from those with early-stage fibrosis. Therefore, the performance of noninvasive tests was primarily evaluated using ROC analyses to distinguish patients with ≥F2 fibrosis (*n* = 33; 58.9% of participants) and ≥F3 fibrosis (*n* = 14; 25% of participants). Nevertheless, due to potential overfitting from the limited sample size, our proposed model warrants cautious interpretation and independent validation in larger cohorts. Second, there were no patients with F0 fibrosis in our cohort. While the absence of F0 cases and the higher-than-expected diagnostic performance of NFS and FIB4 may raise concerns regarding cohort representativeness, the inclusion of all biopsy-confirmed MASH cases during a defined timeframe reflects real-world clinical practice. Third, in addition to identifying the most relevant clinical variables associated with fibrosis stages, we proposed a scoring model that could detect ≥F2 in MASH with greater diagnostic accuracy than existing NITs and MRE. While this model requires validation in both internal and external cohorts, our preliminary findings suggest that future approaches incorporating the total metabolic syndrome score and MMP-1 may hold promise for noninvasive fibrosis staging.

## 5. Conclusions

Given that liver biopsy is not routinely performed in clinical practice and high-quality imaging techniques are often inaccessible or unaffordable, this study evaluated a broad range of clinical variables to identify significant predictors of fibrosis stage in MASH patients. The newly defined “total metabolic syndrome score”, together with MMP-1 level and AST/ALT ratio, can diagnose ≥F2 with a significantly high PPV and also has higher sensitivity and NPV compared to other NITs and MRE kPa. Furthermore, the proposed scoring model achieves the highest specificity (100%) and PPV (100%) in diagnosing advanced fibrosis, with the calculated optimal cut-off value of 2.37.

## Figures and Tables

**Table 1:** Baseline demographic profile, biochemical data, and histological data of the entire cohort.

	F1 (n = 23)	F2 (n = 19)	F3 (n = 6)	F4 (n = 8)	*p* Value
**Demographic Profile**					
Age (year), median (IQR)	43 (33–53)	48 (31–54)	58 (50–62)	63 (54–69)	**0.001**
Female, n (%)	8 (34.8)	9 (47.4)	6 (100.0)	4 (50.0)	**0.042**
BMI (kg/m^2^), median (IQR)	30.57 (27.72–32.40)	33.21 (27.14–35.09)	32.19 (30.86–41.25)	31.01 (29.41–36.05)	0.43
Diabetes mellitus *, n (%)	10 (43.5)	12 (63.2)	5 (83.3)	6 (75.0)	0.21
Hypertension *, n (%)	10 (43.5)	11 (57.9)	5 (83.3)	5 (62.5)	0.35
Dyslipidemia *, n (%)	13 (56.5)	12 (63.2)	5 (83.3)	6 (75.0)	0.59
Metabolic syndrome *, n (%)	11 (47.8)	13 (68.4)	6 (100.0)	8 (100.0)	**0.011**
Total metabolic syndrome score *, median (IQR)	2 (2–3)	4 (3–5)	5 (4–5)	3 (3–5)	**0.001**
**Biochemical Data**					
AST (U/L), median (IQR)	47 (39–68)	54 (39–79)	63 (36–71)	45 (36–52)	0.51
ALT (U/L), median (IQR)	91 (59–133)	109 (68–134)	48 (41–59)	34 (26–39)	**<0.001**
AST/ALT ratio, median (IQR)	0.56 (0.48–0.68)	0.57 (0.50–0.67)	1.26 (1.11–1.41)	1.37 (1.20–1.58)	**<0.001**
GGT (U/L), median (IQR)	59 (43–89)	79 (48–99)	56 (39–141)	130 (68–186)	0.25
Total bilirubin, (mg/dL), median (IQR)	0.65 (0.55–0.93)	0.75 (0.52–0.98)	0.61 (0.54–0.92)	0.97 (0.71–1.28)	0.58
Platelet (×10^3^/mm^3^), median (IQR)	248 (220–314)	257 (232–297)	177 (140–216)	91 (86–114)	**<0.001**
INR, median (IQR)	0.92 (0.90–0.96)	0.95 (0.90–0.97)	1.04 (0.97–1.09)	1.12 (1.07–1.14)	**<0.001**
Albumin (g/dL), median (IQR)	4.70 (4.56–4.83)	4.72 (4.47–4.80)	4.36 (4.05–4.68)	3.91 (3.52–4.11)	**<0.001**
Blood urea nitrogen (mg/dL), median (IQR)	13.0 (10.4–14.5)	12.3 (10.5–14.1)	11.7 (10.7–12.6)	16.6 (14.7–20.4)	**0.035**
HbA1c (%), median (IQR)	6.1 (5.6–7.1)	6.7 (5.8–8.3)	7.6 (5.4–8.9)	6.85 (6.1–7.2)	0.61
Fasting plasma glucose level (mg/dL), median (IQR)	102 (92–133)	112 (97–145)	193 (109–203)	133 (109–178)	**0.020**
LDL (mg/dL), median (IQR)	151 (132–165)	136 (127–150)	138 (122.3–141)	107.5 (86–141.5)	**0.015**
Triglyceride (mg/dL), median (IQR)	158 (114–201)	156.5 (133–201)	183 (153–197)	114 (91–174.5)	0.25
**Histological Data**					
NAFLD activity score (NAS), median (IQR)	4 (3–5)	5 (4–6)	3.5 (3–5)	5 (4–5)	**0.036**

ALT: alanine aminotransferase; AST: aspartate aminotransferase; BMI: body mass index; GGT: gamma-glutamyl transferase; HbA1c: hemoglobin A1c (glycated hemoglobin); INR: international normalized ratio; IQR: interquartile range; LDL: low-density lipoprotein. * The diagnostic criteria used for the recording and analysis of data on comorbidities are summarized in Supplementary Table S1.

**Table 2:** Results of serum fibrosis biomarkers, noninvasive tests and imaging data obtained by MRE and MRI-PDFF.

	F1 (n = 23)	F2 (n = 19)	F3 (n = 6)	F4 (n = 8)	*p* Value
**Serum Fibrosis Biomarkers**					
α2-macroglobulin (g/L), median (IQR)	7.80 (6.00–9.01)	6.80 (6.56–8.57)	7.12 (5.78–9.21)	6.73 (6.20–8.46)	0.83
Apolipoprotein A1 (g/L), median (IQR)	1.01 (0.94–1.22)	1.02 (0.93–1.12)	1.00 (0.88–1.09)	0.97 (0.93–1.04)	0.71
Hyaluronic acid (ng/mL), median (IQR)	797.2 (733.7–1026.3)	749.4 (710.0–937.0)	831.2 (786.0–848.6)	798.2 (756.8–849.9)	0.46
TIMP-1 (ng/mL), median (IQR)	909.2 (812.3–1107.5)	825.1 (718.8–990.5)	844.4 (792.8–922.0)	824.9 (690.4–894.4)	0.18
PIIINP (ng/mL), median (IQR)	25.52 (22.82–28.59)	23.26 (19.45–25.72)	23.43 (22.41–25.18)	24.94 (21.91–26.52)	0.46
MMP-1 (ng/mL), median (IQR)	7.03 (5.05–12.90)	5.85 (4.68–6.71)	6.31 (3.82–6.44)	3.13 (2.72–4.29)	**0.009**
MMP-3 (ng/mL), median (IQR)	17.67 (16.17–20.10)	17.40 (15.33–19.45)	16.84 (15.39–17.26)	17.57 (16.18–18.59)	0.50
**Noninvasive Tests**					
FIB4 score, median (IQR)	0.79 (0.55–1.15)	1.00 (0.59–1.46)	2.16 (1.81–3.78)	5.34 (4.25–5.70)	**<0.001**
NAFLD fibrosis score, median (IQR)	−2.77 (−3.72–−1.22)	−2.14 (−2.64–−0.88)	0.12 (−0.14–2.05)	2.15 (1.68–2.90)	**<0.001**
BARD score, median (IQR)	1 (1–2)	2 (1–2)	4 (4–4)	4 (3–4)	**<0.001**
APRI score, median (IQR)	0.58 (0.41–0.73)	0.68 (0.41–0.90)	0.90 (0.58–1.11)	1.35 (1.10–1.64)	**0.005**
	**F1 (n = 21)**	**F2 (n = 17)**	**F3 (n = 4)**	**F4 (n = 7)**	***p*** **Value**
**Imaging Data**					
MRE kPA, median (IQR)	2.50 (2.40–2.90)	2.70 (2.20–3.50)	4.75 (2.95–6.50)	6.00 (4.00–7.00)	**<0.001**
MRI-PDFF (%), median (IQR)	18.0 (14.5–25.5)	20.0 (16.8–25.3)	11.5 (5.5–18.3)	3.0 (1.0–5.0)	**0.012**

APRI: AST to platelet ratio index; FIB4: Fibrosis-4, IQR: interquartile range; kPa: kilopascal, MMP: matrix metalloproteinase; MRE: magnetic resonance elastography; MRI-PDFF: magnetic resonance imaging-proton density fat fraction; NAFLD: nonalcoholic fatty liver disease; PIIINP: type III procollagen N-terminal propeptide; TIMP: tissue inhibitor of metalloproteinase.

**Table 3:** Results of ROC analysis.

	AUROC (%95 CI)	Optimal Cut-Off	Sensitivity (%)	Specificity (%)	PPV (%)	NPV (%)	*p* Value
**Fibrosis stage ≥ 2**							
New score	0.88 (0.79–0.97)	0.75	77.4	90.9	92.3	74.1	**<0.001**
FIB4 score	0.74 (0.61–0.87)	1.30	60.6	87.0	87.0	60.6	**<0.001**
NAFLD fibrosis score	0.78 (0.66–0.91)	−0.88	61.3	91.3	90.5	63.6	**<0.001**
BARD score	0.69 (0.55–0.83)	3	45.2	87.0	82.4	54.1	**0.008**
APRI score	0.68 (0.54–0.82)	1.00	42.4	95.7	93.3	53.7	**0.014**
MRE kPa	0.73 (0.59–0.88)	3.50	57.1	95.2	94.1	62.5	**0.002**
**Fibrosis stage ≥ 3**							
New score	0.95 (0.90–1.00)	2.37	78.6	100.0	100.0	92.9	**<0.001**
FIB4 score	0.97 (0.94–1.00)	1.81	92.9	92.9	81.3	97.5	**<0.001**
NAFLD fibrosis score	0.99 (0.97–1.00)	−0.46	100.0	90.0	77.8	100.0	**<0.001**
BARD score	0.89 (0.76–1.00)	3	92.9	90.0	76.5	97.3	**<0.001**
APRI score	0.79 (0.63–0.96)	1.00	71.4	88.1	66.7	90.2	**<0.001**
MRE kPa	0.90 (0.77–1.00)	3.50	90.7	81.6	58.8	96.9	**<0.001**

APRI: AST to platelet ratio index; AUROC: area under the receiver operating characteristic; CI: confidence interval; FIB4: Fibrosis-4, kPa: kilopascal, MRE: magnetic resonance elastography; NAFLD: nonalcoholic fatty liver disease; NPV: negative predictive value; PPV: positive predictive value.

## Data Availability

The data presented in this study are available upon reasonable request from the corresponding author due to patient privacy considerations.

## Supplementary Materials

The following supporting information can be downloaded at https://www.mdpi.com/article/10.3390/medicina61061102/s1, Table S1. The diagnostic criteria used for the recording and analysis of data on comorbidities.; Table S2. Blood pressure measurements, anthropometric data and additional biochemical data of the entire cohort.
