# Supplementary material for: Predicting Fibrosis Stage in MASH: The Role of Total Metabolic Syndrome Score and MMP-1
Source: Medicina (Kaunas). 2025 Jun 17;61(6):1102. doi: 10.3390/medicina61061102 (PMC12195011; doi:10.3390/medicina61061102)
Supplement: Supplementary file 1 [file medicina-61-01102-s001.zip › medicina-3690907-supplementary.pdf]

Supplementary Table S1. The diagnostic criteria used for the recording and analysis of data on comorbidities.

**Diabetes Mellitus:** Patients who met at least one of the following criteria were classified as "diagnosed with diabetes", while patients who did not meet any of the following criteria were classified as "not diagnosed with diabetes":	• Fasting plasma glucose level ≥126 mg/dL,<br>• 2-hour plasma glucose level ≥200 mg/dL after a 75 g oral glucose tolerance test,<br>• Random plasma glucose level ≥200 mg/dL in a patient with symptoms of hyperglycemia,<br>• HbA1c ≥6.5%,<br>• Previous diagnosis of diabetes and initiation of treatment based on any of the above criteria.
**Hypertension:** After allowing the patients to rest in a seated position at least 5 minutes, manual blood pressure measurements were taken from both upper extremities, and the higher measurement was recorded. To ensure standardization, all measurements were performed using the same blood pressure measurement device and by the same person. It was ensured that patient had not consumed substances such as cigarettes, coffee, or tea that could affect blood pressure measurements at least one hour prior to the measurements. Patients who met at least one of the following criteria were classified as "diagnosed with hypertension", while those who did not meet any of the following criteria were classified as "not diagnosed with hypertension":	• In office blood pressure measurements, systolic blood pressure ≥140 mmHg and/or diastolic blood pressure ≥90 mmHg,<br>• Previous diagnosis of hypertension based on office measurements, home blood pressure monitoring, and/or ambulatory blood pressure monitoring, and initiation of treatment.
**Dyslipidemia:** Patients who met at least one of the following criteria in lipid profile after a minimum of 8 hours fasting were classified as "diagnosed with hyperlipidemia", while those who did not meet any of the following criteria were classified as "not diagnosed with hyperlipidemia":	• LDL level >190 mg/dL,<br>• LDL level >160 mg/dL with the presence of at least two additional cardiovascular risk factors (At least two of these criteria: Hypertension, family history of premature cardiovascular disease, >65 years old),<br>• LDL level >130 mg/dL with the presence of at least three additional cardiovascular risk factors (All three of these criteria: Hypertension, family history of premature cardiovascular disease, >65 years old),<br>• LDL level >70 mg/dL with the presence of comorbidities such as diabetes mellitus, coronary artery disease, acute coronary syndrome, previous myocardial infarction, history of stroke, peripheral artery disease, carotid artery disease, or abdominal aortic aneurysm,<br>• Triglyceride level >500 mg/dL,<br>• Triglyceride level >200 mg/dL with the presence of comorbidities such as diabetes mellitus, coronary artery disease, acute coronary syndrome, previous myocardial infarction, history of stroke, peripheral artery disease, carotid artery disease, or abdominal aortic aneurysm,<br>• Previous diagnosis of hyperlipidemia and initiation of lipid-lowering therapy based on any of the above criteria.
**Metabolic Syndrome:** Patients who met at least three of the following criteria were classified as "diagnosed with metabolic syndrome", while patients who had fewer than three criteria were classified as "not diagnosed with metabolic syndrome". The number of criteria met by each patient among these five criteria was recorded and included in the analysis as the "total metabolic syndrome score".	• Waist circumference ≥102 cm for men and ≥88 cm for women,<br>• In office blood pressure measurements, systolic blood pressure ≥130 mmHg and/or diastolic blood pressure ≥85 mmHg in patients without a diagnosis of hypertension, or previous diagnosis of hypertension based on office measurements, home blood pressure monitoring, and/or ambulatory blood pressure monitoring with the initiation of antihypertensive medication,<br>• Fasting plasma glucose level ≥100 mg/dL after a minimum of 8 hours of fasting, or previous diagnosis of diabetes with the initiation of treatment,<br>• Fasting triglyceride level ≥150 mg/dL after a minimum of 8 hours of fasting, or previous initiation of lipid-lowering treatment,<br>• HDL level <40 mg/dL in men and <50 mg/dL in a women after a minimum of 8 hours of fasting.

Supplementary Table S2. Blood pressure measurements, anthropometric data and additional biochemical data of the entire cohort.

	**F1 (n=23)**	**F2 (n=19)**	**F3 (n=6)**	**F4 (n=8)**	***p* value**
**Blood Pressure Measurements**					
Systolic blood pressure (mmHg), median (IQR)	130 (120-140)	135 (130-150)	137 (120-145)	142.5 (127.5-150)	0.40
Diastolic blood pressure (mmHg), median (IQR)	80 (75-90)	85 (80-90)	82.5 (76-90)	77.5 (60-80)	0.11
Mean blood pressure (mmHg), median (IQR)	100 (93.3-105)	101.7 (96.7-106.7)	100.2 (93.3-106.7)	97.5 (86.7-103.3)	0.42
**Anthropometric Data**					
Height (cm), median (IQR)	167 (159-174)	167 (164-178)	158.5 (155-165)	165 (153-175)	0.072
Weight (kg), median (IQR)	80.3 (77.2-94.7)	91.1 (80.8-110.0)	86.0 (69.2-99.1)	82.1 (74.7-101.3)	0.47
Waist circumference (cm), median (IQR)	104 (98-114)	112 (98-117)	110.5 (98-119)	112 (105.5-122.5)	0.36
Hip circumference (cm), median (IQR)	106 (103-113)	109 (104-122)	112.5 (107-130)	112.5 (110-117.5)	0.19
Waist-to-hip ratio, median (IQR)	0.97 (0.92-1.02)	0.97 (0.93-0.99)	0.93 (0.90-0.96)	1.0 (0.96-1.04)	0.22
Waist-to-height ratio, median (IQR)	0.63 (0.59-0.67)	0.66 (0.55-0.69)	0.68 (0.66-0.77)	0.68 (0.62-0.76)	0.22
Body fat mass (kg), median (IQR)	23.7 (19.1-30.3)	25.5 (19.9-37.4)	31.8 (22.3-47.6)	25.5 (23.7-31.6)	0.32
Body fat percentage (%), median (IQR)	28.6 (23.0-36.0)	29.3 (26.7-39.4)	37.0 (32.9-48)	33.8 (25.3-36.5)	0.11
Abdominal fat mass (kg), median (IQR)	13.7 (10.5-17.0)	15.1 (10.2-18.5)	14.5 (10.2-21.7)	13.5 (11.7-14.6)	0.98
Ratio of abdominal fat mass/body fat mass, median (IQR)	0.55 (0.46-0.60)	0.52 (0.45-0.60)	0.46 (0.41-0.49)	0.53 (0.44-0.62)	0.31
Fat-free mass (kg), median (IQR)	60.5 (48.9-69.3)	61.4 (55.2-71.6)	50.3 (47.6-51.5)	55.3 (46.8-76.7)	0.18
Ratio of fat-free mass, median (IQR)	0.71 (0.64-0.77)	0.71 (0.61-0.73)	0.63 (0.52-0.67)	0.66 (0.64-0.75)	0.11
Body protein mass (kg), median (IQR)	11.8 (9.9-13.8)	12.2 (11.0-14.0)	10.1 (9.8-10.6)	11.4 (9.7-15.2)	0.17
Body protein percentage (%), median (IQR)	14.6 (12.8-15.1)	14.3 (12.2-15.0)	12.6 (10.7-13.9)	13.7 (13.1-14.9)	0.16
Body mineral mass (kg), median (IQR)	4.3 (3.1-4.7)	4.0 (3.5-5.1)	3.1 (3.0-3.5)	3.4 (2.9-5.1)	0.13
Body mineral percentage (%), median (IQR)	4.7 (4.1-5.5)	4.4 (4.3-4.9)	4.1 (3.2-4.3)	4.1 (4.0-5.1)	0.12
**Biochemical Data**					
ALP (U/L), median (IQR)	88 (76-115)	88 (69-109)	94 (69-118)	96 (79-117)	0.81
Direct bilirubin (mg/dL), median (IQR)	0.12 (0.10-0.16)	0.13 (0.11-0.19)	0.13 (0.10-0.16)	0.23 (0.15-0.31)	0.22
Indirect bilirubin (mg/dL), median (IQR)	0.54 (0.45-0.76)	0.63 (0.42-0.80)	0.49 (0.42-0.75)	0.73 (0.55-1.00)	0.68
aPTT (seconds), median (IQR)	26.0 (24.0-27.3)	24.6 (23.2-26.9)	27.5 (26.2-27.5)	26.2 (24.4-28.9)	0.16
25(OH)D_3 (μg/L), median (IQR)	12.9 (7.5-21.7)	21.0 (12.2-29.5)	23.6 (18.5-29.5)	15.8 (9.8-18.1)	0.25

	F1 (n=23)	F2 (n=19)	F3 (n=6)	F4 (n=8)	*p* value
**Biochemical Data *(continued)***					
HOMA-IR, median (IQR)	4.9 (3.1-7.9)	7.3 (4.0-11.7)	11.7 (8.3-12.6)	6.7 (6.3-11.7)	0.20
Ammonia (µg/dL), median (IQR)	44.3 (23.6-55.1)	45.0 (37.8-54.3)	58.9 (40.4-65.5)	47.1 (37.6-54.4)	0.47
Haptoglobin (mg/dL), median (IQR)	146 (97.3-213)	146.5 (105.5-201)	152 (36.1-178)	65.5 (31.4-113)	0.10
α-fetoprotein (µg/L), median (IQR)	2.9 (2.1-4.1)	2.8 (2.1-5.0)	3.6 (1.6-4.8)	3.8 (2.9-5.1)	0.56
Total cholesterol level (mg/dL), median (IQR)	225 (178-244)	197 (182-228)	201.5 (166-206)	174.5 (135-207.5)	0.11
HDL (mg/dL), median (IQR)	47 (42-57)	40.5 (34-46)	40.5 (39.5-43)	41.5 (36-53.5)	0.11
Non-HDL (mg/dL), median (IQR)	179 (147-192)	156.5 (137-189)	159 (126.5-166)	120.5 (96-166)	0.061

ALP: Alkaline phosphatase; aPTT: Activated partial thromboplastin time; HDL: High-density lipoprotein; HOMA-IR: Homeostatic model assessment for insulin resistance; IQR: Interquartile range; 25(OH)D_3: 25-hydroxyvitamin D_3
